# Aplastic Crisis Triggered by Parvovirus B19 in an Adult Man With Sickle Cell Disease

**DOI:** 10.7759/cureus.110547

**Published:** 2026-06-09

**Authors:** Zehra Rahman, Shelby Watford, Sean Carlson, Brandon Warren, Oday Elmanaseer

**Affiliations:** 1 Department of Internal Medicine, University of Florida College of Medicine – Jacksonville, Jacksonville, USA; 2 Department of Medicine, Idaho College of Osteopathic Medicine, Meridian, USA; 3 Department of Hematology and Medical Oncology, University of Florida College of Medicine – Jacksonville, Jacksonville, USA

**Keywords:** adult sickle cell anemia, aplastic crisis, parvovirus b19, post-splenectomy, sickle cell disease (scd)

## Abstract

Patients with sickle cell disease (SCD) are vulnerable to acute hematologic complications due to chronic hemolysis and dependence on increased erythropoiesis. Parvovirus B19 infection can suppress erythroid progenitor cells, precipitating a transient aplastic crisis characterized by severe anemia and reticulocytopenia. Early recognition is critical to distinguish this condition from other causes of acute anemia in patients with SCD.

A 22-year-old man with hemoglobin SS SCD and prior splenectomy presented with a three-day history of severe headache, non-bloody emesis, fevers, fatigue, and generalized myalgias distinct from his typical vaso-occlusive crises. On presentation, vital signs were stable, and physical examination was largely unremarkable. Laboratory evaluation demonstrated severe anemia with a hemoglobin of 4.5 g/dL (baseline: 7-10 g/dL), leukocytosis (19×10⁹/L), thrombocytopenia (94×10⁹/L), and a reticulocyte count of 2.8%. Hemolysis markers were elevated but consistent with his baseline chronic hemolysis. Peripheral smear revealed 1-3% sickled erythrocytes. Evaluation for alternative causes of acute anemia, including thrombotic microangiopathy, disseminated intravascular coagulation, autoimmune hemolytic anemia, and hemophagocytic lymphohistiocytosis, was unrevealing. Viral studies demonstrated elevated parvovirus B19 IgM and IgG antibodies, confirming acute infection. The patient received four units of packed red blood cells with improvement in hemoglobin to 6.6 g/dL. He was managed with supportive care, and hematologic parameters gradually returned toward baseline prior to discharge.

This case highlights the importance of recognizing parvovirus B19-induced transient aplastic crisis as a potentially life-threatening but treatable complication in patients with SCD. Existing literature describes reticulocytopenia as a key distinguishing feature from other causes of acute anemia in SCD, yet diagnosis may be challenging when patients present with nonspecific viral symptoms or concurrent laboratory evidence of chronic hemolysis. Our case reinforces prior reports emphasizing that an inappropriately low reticulocyte response in the setting of profound anemia should prompt evaluation for parvovirus B19 infection, even in patients with baseline hemolysis and complex hematologic abnormalities. Additionally, the low percentage of sickled erythrocytes on peripheral smear despite severe anemia underscores the importance of considering aplastic crisis rather than vaso-occlusive or hyperhemolytic processes. Early recognition and supportive transfusion therapy remain essential to prevent morbidity and guide appropriate management.

## Introduction

Human parvovirus B19 (*Erythroparvovirus* B19) is a small, non-enveloped, single-stranded DNA virus belonging to the *Parvoviridae* family. The virus has a unique tropism for erythroid progenitor cells due to the presence of the P blood group antigen (globoside) on their surface, which facilitates viral entry. Viral replication within these cells leads to cytotoxicity and the temporary suppression of erythropoiesis [1].

Parvovirus B19 infections remain prevalent worldwide with periodic outbreaks. Infection rates are generally higher in the late winter and spring. In general, individuals are infected at younger ages, often during the school-age years [1]. Parvovirus B19 has a well-known cyclic epidemiology with spikes every 3-4 years [2]. Outbreaks at schools lead to student-to-student, student-to-staff, and staff-to-staff transmission. Although infection is not typically associated with prominent respiratory symptoms, transmission most commonly occurs via respiratory secretions and saliva, and the virus is stable enough to persist on fomites. Though less common, there can also be vertical transmission if a woman acquires the infection during pregnancy, and there is also the possibility for hematogenous transmission through blood transfusions [3].

The common presenting signs of infection are a nonspecific flu-like illness, and children often develop the classic reticulated "slapped cheek" rash. Because parvovirus B19 replicates within and destroys erythroid progenitor cells, acute infection can result in anemia [4]. Patients with underlying hematologic conditions such as sickle cell disease (SCD), thalassemia, or iron deficiency anemia, as well as immunocompromised individuals, can present with severe anemia requiring blood transfusions. These patients often present with fatigue, pallor, weakness, and lethargy. Laboratory findings typically demonstrate severe anemia with absent or inappropriately low reticulocytosis, and bone marrow biopsy typically demonstrates decreased erythroid precursors. This presentation is referred to as a transient aplastic crisis [1]. In patients with SCD, chronic hemolysis necessitates a persistently elevated rate of erythropoiesis; therefore, even transient suppression of erythroid precursors can result in a rapid and severe decline in hemoglobin. Those with hematologic abnormalities usually experience a transient aplastic crisis, whereas immunocompromised hosts often experience pure red cell aplasia [5].

The patient in this case had a history of SCD with inconsistent adherence to hydroxyurea and prior transfusion requirements. He presented with symptoms of a nonspecific viral illness and severe hypoproliferative anemia. Notably, he had multiple recent infections in the month preceding admission, further complicating the diagnostic evaluation. This case highlights the importance of thorough evaluation in distinguishing causes of acute anemia in patients with SCD.

## Case presentation

A 22-year-old man with a past medical history of hemoglobin SS (Hb-SS) SCD and prior surgical splenectomy in childhood presented with a severe headache rated 10/10 in intensity. He reported that the headaches began three days prior to presentation and each episode was preceded by non-bloody emesis. He also endorsed fevers, chills, fatigue, generalized body aches, decreased appetite, and lightheadedness over the preceding several days. He described the body aches as generalized myalgias, noting that they were different from his typical vaso-occlusive pain crises. He denied chest pain, shortness of breath, or diarrhea. He was prescribed hydroxyurea as an outpatient, but reported non-adherence for several months prior to hospital admission. He denied recent use of other medications known to contribute to bone marrow suppression or exacerbate aplastic crisis.

On presentation, his vital signs were as follows: blood pressure 114/57 mmHg, heart rate 85 beats per minute, temperature 99.2°F (37.3°C), respiratory rate 13 breaths per minute, and oxygen saturation 93% on room air. The physical exam was largely unremarkable, without wheezing, external bleeding, or rashes. He appeared fatigued and lethargic but was otherwise in no acute distress. Head imaging was unremarkable for any acute process, and other imaging revealed no acute bleeding or abnormalities.

**Figure 1:**
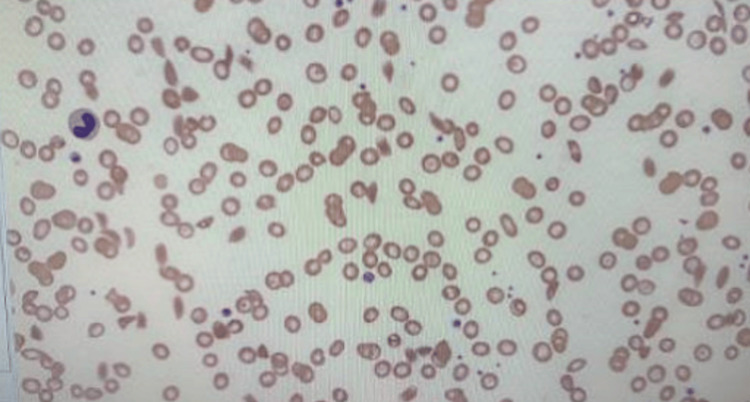
Peripheral blood smear showing sickled erythrocytes consistent with hemoglobin SS sickle cell disease. No schistocytes or other abnormal red cell morphologies are noted. Wright-Giemsa stain: 1000×

Initial laboratory studies were notable for a severe borderline microcytic anemia with a hemoglobin of 4.5 g/dL and a mean corpuscular volume (MCV) of 77.2 fL compared to a baseline hemoglobin of 7-10 g/dL. There was a leukocytosis of 19×10⁹/L with 64% lymphocytes, as well as thrombocytopenia with a platelet count of 94×10⁹/L. Peripheral smear demonstrated sickle erythrocytes without polychromasia (Figure 1). 

Hemolytic evaluation revealed an elevated total bilirubin of 3.9 mg/dL, direct bilirubin of 1.0 mg/dL, haptoglobin of <10 mg/dL, lactate dehydrogenase (LDH) of 880 IU/L, and a reticulocyte count of 2.8%, representing an inappropriately low response relative to the degree of anemia. Notably, prior reticulocyte counts were significantly higher at 15.9% one month before admission and 12.1% one year prior. In the setting of chronic hemolysis and expected compensatory reticulocytosis in SCD, this relative reticulocytopenia raised concern for superimposed bone marrow suppression (Table 1).

**Table 1:** Laboratory values demonstrating severe anemia, reticulocytopenia, and hemolytic markers in a patient with sickle cell disease at hospital admission, one year and one month prior to admission, and at discharge

Hemolysis labs	1 year prior	1 month prior	On admission	On discharge	Reference range
Hemoglobin (g/dL)	8.9	8.8	4.5	6.6	14.0-18.0
Reticulocyte count (%)	12.1	15.9	2.8	5.1	0.5 to <1.5
Lactate dehydrogenase (IU/L)	274	264	880	511	126-266
Total bilirubin (mg/dL)	2.5	3.4	3.9	0.7	0.2-1
Haptoglobin (mg/dL)	<10	<10	<10	<10	30-200

Vitamin B12 and folate levels were within normal limits. Iron panel demonstrated markedly elevated ferritin level (5,794 ng/mL; reference range: ~24-336 ng/mL), elevated serum iron (205 µg/dL; reference range: ~60-170 µg/dL), and transferrin saturation (97%; reference range: ~20-50%), with a low total iron-binding capacity (TIBC) (166 µg/dL; reference range: ~240-450 µg/dL). In the setting of acute illness, these findings are most consistent with inflammation-associated dysregulation of iron homeostasis, as ferritin is an acute-phase reactant and transferrin is a negative acute-phase protein. In patients with SCD, this pattern may also reflect underlying iron overload from chronic hemolysis and prior transfusion exposure, which is further exacerbated during acute hospitalizations requiring red blood cell transfusion. Although markedly elevated ferritin can be seen in conditions such as hemophagocytic lymphohistiocytosis, the overall clinical and laboratory evaluation in this case did not support this diagnosis. Aspartate aminotransferase (AST) was 64 U/L, and triglycerides were 92 mg/dL, decreasing suspicion for hemophagocytic lymphohistiocytosis. Hemoglobin electrophoresis demonstrated hemoglobin S at 75%, consistent with his known Hb-SS diagnosis. Hemoglobin F was 15.3%, consistent with prior hydroxyurea therapy.

A direct antiglobulin test was positive; however, C3d testing and eluate antibody testing were negative, findings not consistent with warm autoimmune hemolytic anemia. Fibrinogen was within normal limits at 282 mg/dL, making disseminated intravascular coagulation unlikely. Peripheral smear did not demonstrate schistocytes, and ADAMTS13 activity was within normal limits, decreasing suspicion for thrombotic thrombocytopenic purpura or hemolytic uremic syndrome.

Of note, the patient had a recent hospital admission within the past few weeks, where he tested positive for respiratory syncytial virus (RSV) and group A *Streptococcus*, for which he received treatment including hydroxyurea and was discharged with a seven-day course of amoxicillin-clavulanate.

Viral testing was negative for Epstein-Barr virus (EBV), cytomegalovirus (CMV), and human immunodeficiency virus (HIV). Hepatitis serologies demonstrated positive for hepatitis B core IgM antibody and hepatitis B surface antibody, negative for hepatitis B surface antigen, and undetectable hepatitis B DNA, consistent with prior resolved hepatitis B infection with immunity. 

Further viral testing demonstrated elevated parvovirus B19 IgM antibody (21.2 index value) and IgG antibody (2.4 index value), consistent with acute parvovirus infection with developing immunity. In the setting of severe anemia and an inappropriately low reticulocyte response, these findings raised concern for parvovirus-induced aplastic crisis.

The patient received four units of packed red blood cells, resulting in an improvement of hemoglobin to 6.6 g/dL. A bone marrow biopsy was performed, which showed hypercellular bone marrow (90%) with a special stain for parvovirus highlighting scattered positive cells. It was negative for increased blasts and significant dysplasia. Peripheral blood and bone marrow flow cytometry were negative for increased blasts, monotypic B-cell populations, plasma cell proliferation, or aberrant T-cell populations. These findings helped exclude other causes of cytopenias and supported parvovirus B19-associated transient aplastic crisis as the primary etiology of the patient's presentation. While bone marrow biopsy is not routinely required for the diagnosis of parvovirus-induced aplastic crisis, it may be valuable in atypical presentations when alternative marrow disorders remain under consideration.

The patient was managed with supportive care for parvovirus infection, with close monitoring of hemolytic markers. Transfusions were administered as needed to maintain a hemoglobin threshold of >6.5 g/dL. Over the course of hospitalization, his hemolytic markers trended toward his baseline chronic hemolytic levels, and he was safely discharged. Hydroxyurea was held during hospitalization, and the patient was scheduled for hematology outpatient follow-up to restart hydroxyurea after the resolution of the aplastic crisis.

## Discussion

Patients with SCD are susceptible to a variety of acute hematologic complications due to chronic hemolysis, functional asplenia, and bone marrow stress from ongoing erythropoiesis. Among these complications, transient aplastic crisis secondary to parvovirus B19 infection represents a well-described but potentially life-threatening complication, particularly in individuals with underlying hemolytic anemias [6]. In these patients, erythrocyte survival is significantly shortened, and maintenance of baseline hemoglobin levels depends on a continuously elevated rate of erythropoiesis. Infection with parvovirus B19 disrupts this compensatory mechanism by directly suppressing erythroid precursor cells in the bone marrow, leading to an abrupt cessation of red blood cell production [7].

The classic presentation of parvovirus-induced aplastic crisis in SCD includes acute severe anemia with a markedly decreased or absent reticulocyte response, often accompanied by nonspecific viral symptoms such as fever, malaise, headache, or myalgias. In the present case, the patient demonstrated a profound drop in hemoglobin from his baseline of 7-10 g/dL to 4.5 g/dL, with an inappropriately low reticulocyte count of 2.8% despite severe anemia. This relative reticulocytopenia was a key diagnostic clue suggesting impaired marrow erythropoiesis rather than increased hemolysis alone [8]. Although laboratory studies demonstrated elevated LDH, indirect hyperbilirubinemia, and low haptoglobin consistent with hemolysis, these findings were similar to the patient's baseline chronic hemolytic profile and therefore did not fully explain the severity of his anemia.

The differential diagnosis for acute anemia with thrombocytopenia and leukocytosis in patients with SCD is broad and includes hyperhemolysis syndrome, splenic or hepatic sequestration, autoimmune hemolytic anemia, thrombotic microangiopathy, disseminated intravascular coagulation, and hemophagocytic lymphohistiocytosis. In this case, several diagnostic considerations were systematically excluded. The absence of schistocytes on peripheral smear and normal ADAMTS13 activity decreased suspicion for thrombotic thrombocytopenic purpura or other thrombotic microangiopathies. Normal fibrinogen levels and lack of coagulopathy made disseminated intravascular coagulation unlikely. While the direct antiglobulin test was positive, the absence of C3d and negative eluate testing did not support warm autoimmune hemolytic anemia. Additionally, the patient's normal triglyceride and fibrinogen levels reduced concern for hemophagocytic lymphohistiocytosis. Ultimately, the presence of positive parvovirus B19 IgM antibodies combined with reticulocytopenia and severe anemia supported the diagnosis of parvovirus-induced aplastic crisis.

Another notable feature of this case was the patient's history of surgical splenectomy, which may have contributed to his susceptibility to viral infection and hematologic complications. Functional or anatomic asplenia in SCD impairs immune clearance of pathogens and may predispose patients to more severe infections. Additionally, the patient reported non-adherence to hydroxyurea therapy, which is known to reduce the frequency of SCD-related complications by increasing fetal hemoglobin levels and decreasing hemolysis. Hydroxyurea may also mitigate the severity of anemia by improving overall erythrocyte survival, and lapses in therapy could have further exacerbated the severity of this patient's presentation.

Management of parvovirus B19-associated aplastic crisis is largely supportive, as the infection is typically self-limited in immunocompetent individuals [9]. The primary intervention involves red blood cell transfusion to maintain adequate oxygen-carrying capacity while bone marrow erythropoiesis recovers. In most patients, reticulocytosis resumes within approximately 7-10 days following the onset of infection as viral replication declines and immune-mediated clearance occurs [10]. In this case, transfusion of packed red blood cells resulted in the stabilization of hemoglobin levels, and the patient's hemolytic markers gradually trended towards his baseline hemolytic state prior to discharge.

This case highlights several important clinical considerations in the evaluation of acute anemia in patients with SCD. First, reticulocyte counts must be interpreted in the context of baseline hemolysis, as a seemingly modest reticulocyte percentage may actually represent an inadequate marrow response in the setting of severe anemia. Second, parvovirus B19 infection should remain high on the differential diagnosis when patients with SCD present with sudden severe anemia and reticulocytopenia, particularly when preceded by viral prodromal symptoms. 

Transient aplastic crisis occurs in a substantial proportion of patients with SCD following parvovirus B19 infection, reflecting their reliance on high baseline erythropoietic activity [11]. Early recognition of this complication is critical to ensure timely supportive management and prevent potentially life-threatening sequelae.

Failure to recognize and promptly manage parvovirus B19-associated aplastic crisis in patients with SCD can result in significant morbidity and potentially life-threatening complications. Because patients with SCD rely on increased baseline erythropoiesis to compensate for chronic hemolysis, even a temporary interruption in red blood cell production can lead to profound anemia and critical reductions in oxygen-carrying capacity. Untreated severe anemia may result in high-output cardiac failure, symptomatic hypoxia, syncope, end-organ ischemia, and hemodynamic instability. Additionally, delayed diagnosis may lead to unnecessary investigations for alternative hematologic disorders and the postponement of appropriate transfusion support. In severe cases, prolonged tissue hypoxia may precipitate vaso-occlusive complications, acute chest syndrome, or multi-organ dysfunction. Early recognition of reticulocytopenia and timely supportive transfusion are therefore essential to prevent adverse outcomes and facilitate recovery while bone marrow erythropoiesis resumes.

This case adds to the existing literature by illustrating the diagnostic challenges that may arise when parvovirus B19-associated aplastic crisis presents with concomitant hematologic abnormalities beyond isolated anemia and reticulocytopenia. The presence of thrombocytopenia, leukocytosis, chronic baseline hemolysis, and a positive direct antiglobulin test initially raised concern for several competing diagnoses. Our report highlights the importance of interpreting reticulocyte counts relative to the severity of anemia and baseline hemolytic status in patients with SCD. Furthermore, it demonstrates how systematic exclusion of alternative causes of acute cytopenias, combined with targeted viral testing, can facilitate prompt recognition of aplastic crisis and appropriate supportive management.

Overall, this case underscores the importance of maintaining a broad differential diagnosis in patients with SCD presenting with acute anemia and demonstrates the diagnostic value of correlating reticulocyte responses, hemolytic markers, and viral testing to distinguish aplastic crisis from other hematologic complications of SCD.

## Conclusions

Parvovirus B19 infection should be strongly considered in patients with SCD who present with acute severe anemia and reticulocytopenia. Early recognition allows for prompt supportive management and avoidance of unnecessary diagnostic interventions. This case highlights the importance of interpreting reticulocyte responses in the context of baseline hemolysis when evaluating acute anemia in patients with SCD.
